# Doppler Modeling and Simulation of Train-to-Train Communication in Metro Tunnel Environment

**DOI:** 10.3390/s22114289

**Published:** 2022-06-04

**Authors:** Pengyu Zhao, Xiaoyong Wang, Kai Zhang, Yanliang Jin, Guoxin Zheng

**Affiliations:** 1 Key Laboratory of Specialty Fiber Optics and Optical Access Networks, Joint International Research Laboratory of Specialty Fiber Optics and Advanced Communication, Shanghai Institute for Advanced Communication and Data Science, Shanghai University, Shanghai 200444, China; 18321445156@163.com (P.Z.); gxzheng@staff.shu.edu.cn (G.Z.); 2 CASCO Signal Ltd., Shanghai 200071, China; wangxiaoyong@casco.com.cn; 3 Research Institute of Intelligent Networks, Zhejiang Lab, Hangzhou 311121, China; 13262733921@163.com

**Keywords:** train-to-train (T2T) communication, doppler spread, channel simulation, metro tunnel

## Abstract

The communication system of urban rail transit is gradually changing from train-to-ground (T2G) to train-to-train (T2T) communication. The subway can travel at speeds of up to 200 km/h in the tunnel environment, and communication between trains can be conducted via millimeter waves with minimum latency. A precise channel model is required to test the reliability of T2T communication over a non-line-of-sight (NLoS) Doppler channel in a tunnel scenario. In this paper, the description of the ray angle for a T2T communication terminal is established, and the mapping relationship of the multipath signals from the transmitter to the receiver is established. The channel parameters including the angle, amplitude, and mapping matrix from the transmitter to the receiver are obtained by the ray-tracing method. In addition, the channel model for the T2T communication system with multipath propagations is constructed. The Doppler spread simulation results in this paper are consistent with the RT simulation results. A channel physics modelling approach using an IQ vector phase shifter to achieve Doppler spread in the RF domain is proposed when paired with the Doppler spread model.

## 1. Introduction

With the development of vehicle-to-everything (V2X) communication technology, intelligent transportation systems consisting of vehicle-to-vehicle (V2V) communication, vehicle-to-infrastructure (V2I) communication, vehicle-to-pedestrian (V2P) communication, and vehicle-to-cloud network (V2N) communication have been introduced [1,2]. V2X communication technology mainly includes dedicated short-range communication (DSRC)-based vehicle networks and cellular-based vehicle networks [3]. DSRC is a wireless access solution based on the IEEE 802.11p standard with good distributed characteristics but shortcomings including a limited coverage, low transmission rate, and inability to be used in high-speed mobile environments. The cellular network-based wireless access technology C-V2X developed by the Third Generation Partnership Project (3GPP) enables vehicles to operate normally in scenarios with and without cellular network coverage [4]. In order to improve the performance of V2X communication systems, 3GPP has successively released the LTE-V2X network based on Long Term Evolution (LTE) and a 5G-V2X network for fifth-generation new radio (5G NR) [5]. However, as the number of communication devices and digital applications grows, communication systems face new challenges in terms of data rate, latency, coverage, spectral efficiency, and security [6,7,8]. Industry 5.0 will have access to sixth-generation (6G) wireless communication networks with data rates up to 0.1 TB/s and massive ultra-high-speed wireless access [9]. Wireless data sharing between entities such as vehicles, trains, drones, infrastructure roadside units, traffic data centers, etc., can be further achieved with industry 5.0 [10]. Combine artificial intelligence, cloud computing, block chain, and other emerging technologies to develop reliable autonomous driving applications [11], reasonably plan vehicle routes and driving speeds, reduce vehicle energy consumption and promote green development of traffic.

### 1.1. Related Literature

In the Orthogonal Multiple Access (OMA) technology used by existing networks, the limitation of spectrum resources results in low access efficiency and low data rates in dense vehicular networks. In the next generation of V2X communication, the efficient utilization of radio resources by the multiplexing of transmitted signals and successive interference cancellation (SIC) at the receiving end by using the Non-Orthogonal Multiple Access (NOMA) technique is proposed [12]. Reference [13] proposed a two-stage scheme of centralized resource allocation and distributed power control strategy that increased communication capacity by 5% while lowering transmission power usage by 36%. Reference [14] also verified that the system capacity can be improved by adopting centralized resource allocation and distributed power control methods. In [15], a roadside unit-based energy-saving power allocation scheme for NOMA was proposed, which analyzed the energy efficiency maximization problem under the constraints of incomplete channel state information (CSI), quality of service (QoS), and power constraints. Reference [16] proposed a cognitive radio-assisted NOMA-V2X system model and analyzed its outage probability and throughput performance. Reference [17] provided a new approach to improve spectrum utilization and system capacity, and it also addressed the near–far problem of NOMA-enabled backscatter communication systems. Reference [18] proposed an energy-efficient transmission optimization framework for V2X networks based on ambient backscatter communication and NOMA for imperfect CSI. Under the premise of ensuring QoS, the total transmit power of the V2X network is minimized by optimizing the power distribution of the base station and the reflection coefficient of the backscatter sensor. Compared with the traditional NOMA-based V2X network, the V2X network using the fusion of ambient backscatter communication and NOMA has advantages in terms of energy efficiency. On the other hand, the deep integration of artificial intelligence (AI) tools with wireless communication systems [19,20,21] can further improve network performance [22,23] and plays an important role in channel estimation, scene recognition, velocity prediction, channel characteristic prediction [24,25,26], and channel modeling [27,28,29]. The control system based on T2T communication extends the T2G wireless communication system between trains. The front and rear trains can interchange data immediately, allowing the back train to acquire real-time position and speed information from the front train. As a result, railway operations can be made safer and the safe spacing between trains can be reduced. LTE wireless communication technology has the advantages of having strong anti-interference ability, a high transmission rate, and low system delay. It can be further applied to the train control system based on T2T communication [30]. However, the Doppler shift induced by a high train speed and high signal frequency will have an impact on the wireless communication system’s performance.

### 1.2. Motivation and Contributions

To verify the T2T communication’s reliability, the T2T communication channel model must be implanted into a simulation platform of a physical channel with time varying traffic, and the train control system must be combined to form a hardware-in-the-loop simulation platform to complete the T2T communication performance verification. Therefore, it is necessary to establish a channel simulation model of T2T communication, especially the Doppler spread model caused by high-speed movement. Existing Doppler research on T2T communication mainly focuses on ground scenarios [31,32,33,34,35,36] and V2V communication [37] scenarios in highway tunnels. However, the subway tunnel is narrow and long, including straight, curved, climbing, descending, and other scenarios, making it more challenging than the ground tunnel. Trains using millimeter wave communications traveling at high speeds of more than 200 km/h along a given track in a tunnel environment cause higher Doppler shifts. Thus far, the channel modeling work in subway tunnels has mainly studied the influence of the LoS path, and the single-bounced and double-bounced signal on the channel in the same coordinate space in the V2G communication scene [38,39,40]. In the T2T communication system, the transceiver ends are located in different locations, so it is difficult to apply the same coordinate space analysis. In addition, the movement of the antenna causes rapid changes in the channel environment, complicating the signal propagation process. The influence of multi-bounced signals and the movement of the transceiver antenna on the Doppler shift must be considered, so a Doppler spread model suited for the movement of two-terminal terminals in the tunnel must be established.

In this paper, the receiver (Rx) and transmitter (Tx) coordinate systems are established, and the Doppler spread model of the multipath signals from the transmitter to the receiver is established in their respective coordinate systems. The mapping matrix approach is proposed as an innovative solution to the problem of signal matching at the receiver and transmitter, as well as a method for obtaining the mapping matrix. In order to verify the proposed Doppler model for T2T communication in the tunnel, the ray-tracing (RT) [41] approach is utilized to obtain the angle and amplitude of the signals at the transmitting and receiving ends. By comparing the simulation results of the RT approach with the simulation results of the Doppler spread model, the validity of the Doppler spread model is proved. A channel physical simulation method using IQ vector phase shifters [42,43] is proposed to execute the T2T communication channel simulation in the tunnel environment, which can be used for future tunnel environments when combined with the T2T channel model and test data analysis in the tunnel [44,45,46]. It can provide a reference for 5G millimeter wave physical channel simulation in the future tunnel environment.

### 1.3. Article Structure

The rest of this paper is organized as follows: In Section 2, the Doppler shift models for transmitted and received signals are established, followed by the solution method for the multipath signal mapping relationship at the transmit and receive ends and the multipath signal’s Doppler spread model. In Section 3, the RT simulation method is used to obtain the angle, amplitude, and mapping relationship of the transmitted and received signals, and the Doppler spread simulation of the theoretical model is discussed. In Section 4, combined with the analysis of the communication signal in the tunnel, a method to realize physical channel simulation using an IQ vector phase shifter is proposed. Finally, Section 5 provides the conclusion of this paper.

## 2. Modeling of Wireless Channel for T2T Communication in Tunnel Scenario

### 2.1. Wireless Channel Model of T2T Communication

**Figure 1:**
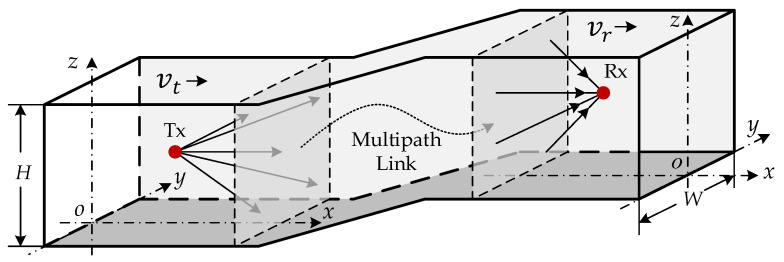
Three-dimensional map of T2T communication in tunnel scenario.

Figure 1 depicts a T2T communication scenario in which the front and rear trains run through a tunnel with a width W and a height H. The complicated propagation process of multipath signals through straight or curved tunnels, such as reflection and scattering, is represented by the multipath link between two trains. The space where the multipath link is located represents the complex channel environment, such as straight or curved tunnels. A three-dimensional coordinate system is established at the transceiver antennas, with the tunnel depth as the *x*-axis, the height as the *z*-axis, and the horizontal direction as the *y*-axis. The downlink of the T2T communication consists of the transmitting antenna Tx located in the rear train and the receiving antenna Rx located in the front train. In addition, $v_{t}$ and $v_{r}$ represent the moving speed of the transmitting antenna and the receiving antenna, and the moving direction follows the same path as the *x*-axis. The received signal consists of the NLoS signal that reaches the receiving antenna after multiple reflections and scattering. In the LoS scenario, it includes the LoS signal from the transmitting antenna to the receiving antenna. In order to study the doppler spread of T2T communication in the tunnel environment, it is assumed that the receiving and the transmitting antennas are omnidirectional uniform antennas.

**Figure 2:**
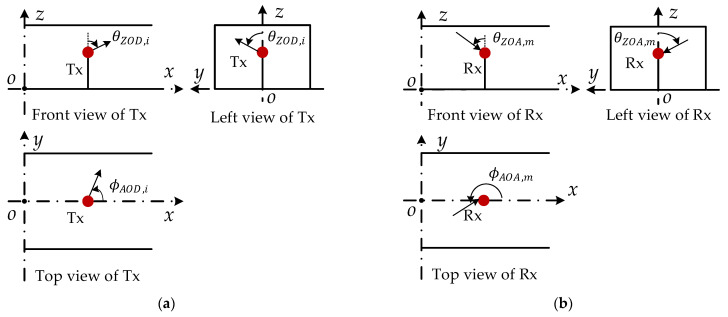
Three views of T2T communication scenario: (**a**) transmitter, (**b**) receiver.

Figure 2 shows the schematic diagram of the signal leaving the transmitting antenna and arriving at the receiving antenna. $\theta_{ZOD,i}$ and $\phi_{AOD,i}$ represent the zenith angle of departure (ZOD) and the azimuth angle of departure (AOD) of the *i*-th transmitted signal. $\theta_{ZOA,m}$ and $\phi_{AOA,m}$ represent the zenith angle of arrival (ZOA) and azimuth angle of arrival (AOA) of the *m*-th received signal, where $\theta_{ZOD,i},\theta_{ZOA,m}\in \left[0,180{}^{\circ}\right]$, $\phi_{AOD,i},\phi_{AOA,m}\in \left[0{}^{\circ},360{}^{\circ}\right)$, 1≤i≤I, 1≤m≤M, and I=M, I and M represent the number of multipaths of transmitted and receiving signals. Let $P_{t,i}$ denote the transmit power of the *i*-th path signal, and $P_{r,m}$ denote the receive power of the *m*-th path signal. Then the spherical coordinate form of the transmitter signal and the receiver signal can be expressed as $\left(P_{t,i},\theta_{ZOD,i},\phi_{AOD,i}\right)$ and $\left(P_{r,m},\theta_{ZOA,m},\phi_{AOA,m}\right)$. Due to the movement of the transmitting antenna, the Doppler shift $f_{d,t}^{i}$ of the signal $\left(P_{t,i},\theta_{ZOD,i},\phi_{AOD,i}\right)$ can be expressed as

(1) $$f_{d,t}^{i}=\frac{v_{t}}{\lambda_{0}}\cos\left(\psi_{t,i}\right)$$

(2) $$\cos\left(\psi_{t,i}\right)={\hat{r}}_{t,i}^{T}\cdot {\hat{v}}_{t}$$

(3) $${\hat{r}}_{t,i}=\left[\begin{array}{c} \sin\left(\theta_{ZOD,i}\right)\cos\left(\phi_{AOD,i}\right)\\ \sin\left(\theta_{ZOD,i}\right)\sin\left(\phi_{AOD,i}\right)\\ \cos\left(\theta_{ZOD,i}\right) \end{array}\right]$$

(4) $${\hat{v}}_{t}={\left[1,0,0\right]}^{T}$$

where $\psi_{t,i}$ represents the angle between the signal $\left(P_{t,i},\theta_{ZOD,i},\phi_{AOD,i}\right)$ and the moving direction of Tx. ${\hat{r}}_{t,i}$ and ${\hat{v}}_{t}$ represent the unit vector in the direction of the transmitted signal and the unit vector in the direction of Tx movement. $\lambda_{0}$ is the signal carrier wavelength, and ${\left(\cdot \right)}^{T}$ represents the transpose of the matrix. Due to the movement of the receiving antenna, the Doppler shift $f_{d,r}^{m}$ of the signal $\left(P_{r,m},\theta_{ZOA,m},\phi_{AOA,m}\right)$ at the receiving end can be expressed as

(5) $$f_{d,r}^{m}=\frac{v_{r}}{\lambda_{0}}\cos\left(\psi_{r,m}\right)$$

(6) $$\cos\left(\psi_{r,m}\right)={\hat{r}}_{r,m}^{T}\cdot {\hat{v}}_{r}$$

(7) $${\hat{r}}_{r,m}=\left[\begin{array}{c} \sin\left(\theta_{ZOA,m}\right)\cos\left(\phi_{AOA,m}\right)\\ \sin\left(\theta_{ZOA,m}\right)\sin\left(\phi_{AOA,m}\right)\\ \cos\left(\theta_{ZOA,m}\right) \end{array}\right]$$

(8) $${\hat{v}}_{r}={\left[1,0,0\right]}^{T}$$

where $\psi_{r,m}$ represents the angle between the signal $\left(P_{r,m},\theta_{ZOA,m},\phi_{AOA,m}\right)$ and the moving direction of Rx. ${\hat{r}}_{r,m}$ and ${\hat{v}}_{r}$ represent the unit vector of the received signal direction and the unit vector of the Rx moving direction.

### 2.2. Matching of Receiving and Transmitting Rays

**Figure 3:**
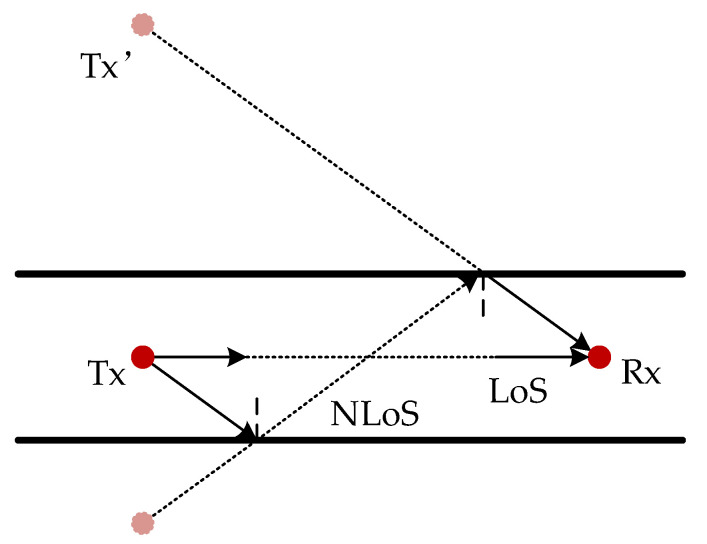
Channel model based on spatial mirror method.

In the proposed system, the transmitted and the received signals have a one-to-one mapping relationship, which means that for each received signal, a unique corresponding transmitted signal can always be located, completing a full signal chain from Tx to Rx. According to the roughness of the sidewall of the tunnel, the mapping relationship between the transmitted and receiving signals can be solved by the spatial mirror method and the random scatterer distribution method. The space mirror method is shown in Figure 3.

For the NLoS propagation path in the tunnel, this method solely considers reflection, and the mirror space is formed with the reflection surface as the axis. For the signal reflected for *k* (*k* = 0, 1, 2, …, *K*) times, the mirror image point Tx’ of transmitting antenna Tx needs to be obtained through *k* times of mirror image.

**Figure 4:**
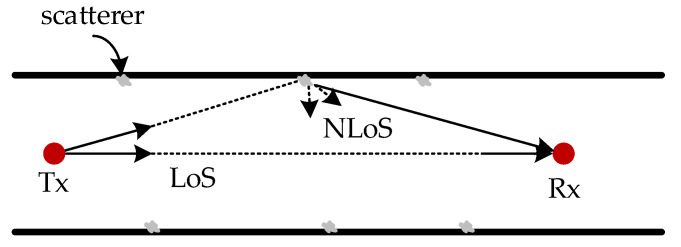
Channel model based on geometric space random scatter.

The scatterers’ location at the tunnel’s size wall follows a uniform random distribution, as shown in Figure 4. In this model, scatterers are randomly distributed along the inner wall of the tunnel. When the NLoS signal passes through the scatterer, the rough scatterer surface leads to a certain randomness in the direction of the secondary radiation wave. When the signal is repeatedly scattered, the mapping between the transmitted signal and the received signal can be considered as random mapping.

The mapping relationship between the transmitter multipath signal and the receiver multipath signal can be expressed as

(9) $$\Gamma =\left(\begin{array}{c} \Gamma_{1}\\ \Gamma_{2}\\ \vdots \\ \Gamma_{I} \end{array}\right)=AN^{T}=\left(\begin{array}{c} \begin{array}{cc} \begin{array}{cc} a_{11} & a_{12} \end{array} & \begin{array}{cc} \cdots & a_{1M} \end{array} \end{array}\\ \begin{array}{cc} \begin{array}{cc} a_{21} & a_{22} \end{array} & \begin{array}{cc} \cdots & a_{2M} \end{array} \end{array}\\ \begin{array}{cc} \begin{array}{cc} \vdots & \vdots \end{array} & \begin{array}{cc} \ddots & \vdots \end{array} \end{array}\\ \begin{array}{cc} \begin{array}{cc} a_{I1} & a_{I2} \end{array} & \begin{array}{cc} \cdots & a_{IM} \end{array} \end{array} \end{array}\right)\left(\begin{array}{c} 1\\ 2\\ \vdots \\ M \end{array}\right)$$

(10) $$A={\left(\begin{array}{c} \begin{array}{cc} \begin{array}{cc} a_{11} & a_{12} \end{array} & \begin{array}{cc} \cdots & a_{1M} \end{array} \end{array}\\ \begin{array}{cc} \begin{array}{cc} a_{21} & a_{22} \end{array} & \begin{array}{cc} \cdots & a_{2M} \end{array} \end{array}\\ \begin{array}{cc} \begin{array}{cc} \vdots & \vdots \end{array} & \begin{array}{cc} \ddots & \vdots \end{array} \end{array}\\ \begin{array}{cc} \begin{array}{cc} a_{I1} & a_{I2} \end{array} & \begin{array}{cc} \cdots & a_{IM} \end{array} \end{array} \end{array}\right)}_{I\times M}$$

(11) $$N={\left(\begin{array}{cc} \begin{array}{cc} 1 & 2 \end{array} & \begin{array}{cc} \cdots & M \end{array} \end{array}\right)}_{1\times M}$$

where *A* is the mapping matrix with *I* rows and *M* columns. The element $a_{im}$ in the mapping matrix represents the mapping relationship between the signal $\left(P_{r,m},\theta_{ZOA,m},\phi_{AOA,m}\right)$ at the receiving end and the signal $\left(P_{t,i},\theta_{ZOD,i},\phi_{AOD,i}\right)$ at the transmitting end. Let the transmitter signal $\left(P_{t,x},\theta_{ZOD,x},\phi_{AOD,x}\right)$ and the receiver signal $\left(P_{r,y},\theta_{ZOA,y},\phi_{AOA,y}\right)$ be the same signal, where 1≤x≤M, 1≤y≤M*,* then the element $a_{im}$ is

(12) $$a_{im}=\{\begin{array}{c} 1,\;i=x,m=y\\ 0,\;i=x,m\neq y \end{array}$$

The matrix ***N*** is the subscript matrix of the multipath signal at the receiving end. After rearranging the mapping matrix, the subscript matrix Γ corresponding to the signal at the transmitting end is obtained.

### 2.3. Doppler Effect at the Transmitter and Receiver

Rearrange the Doppler shifts of each ray at the receiver so that they are in the same order as the corresponding ray at the transmitter

(13) $$ff_{d,r}^{i}=f_{d,r}^{\Gamma_{i}}$$

When both the receiving and transmitting ends move, the Doppler shift $f_{d}^{i}$ of the communication signal can be expressed as

(14) $$f_{d}^{i}=f_{d,t}^{i}+ff_{d,r}^{i}$$

Assuming that the transmitted signal is $x_{p}\left(t\right)=\cos\left(2\pi f_{c}t\right)$, the bandpass form $R_{p}^{i}\left(t\right)$ of the *i*-th path received signal can be expressed as

(15) $$R_{p}^{i}\left(t\right)=c_{i}\cos\left[2\pi \left(f_{c}+f_{d}^{i}\right)\left(t-\tau_{i}\right)\right]$$

where $c_{i}$ and $\tau_{i}$ represent the power normalized amplitude and time delay of the *i*-th path signal, so the baseband form $R_{b}^{i}\left(t\right)$ of the *i*-th path received signal is expressed as

(16) $$R_{b}^{i}\left(t\right)=c_{i}e^{j2\pi \left(f_{d}^{i}t-f_{d}^{i}\tau_{i}-f_{c}\tau_{i}\right)}=c_{i}e^{-j\varphi_{i}}e^{j2\pi f_{d}^{i}t}$$

where $\varphi_{i}=2\pi \left(f_{d}^{i}\tau_{i}+f_{c}\tau_{i}\right)$, when $f_{c}\gg f_{d}^{i}$, $\varphi_{i}\approx 2\pi f_{c}\tau_{i}$; thus, the baseband form of the multipath received signal can be expressed as

(17) $$R_{b}\left(t\right)=\overset{I}{\underset{i=1}{\sum }}c_{i}e^{-j\varphi_{i}}e^{j2\pi f_{d}^{i}t}$$

When the number of rays I→∞, the received signal can be expressed as the integral function of all frequency components from the minimum Doppler frequency $f_{d,min}$ to the maximum Doppler frequency $f_{d,max}$

(18) $$R_{b}\left(t\right)=\overset{f_{d,max}}{\underset{f_{d,min}}{\int }}\sqrt{P\left(f_{d}\right)}e^{-j\varphi_{i}}e^{j2\pi f_{d}t}\;df_{d}$$

where $P\left(f_{d}\right)$ represents the continuous Doppler spectral function.

## 3. Doppler Spread Simulation and Results

### 3.1. Simulation Model and Parameter Settings of RT

**Figure 5:**
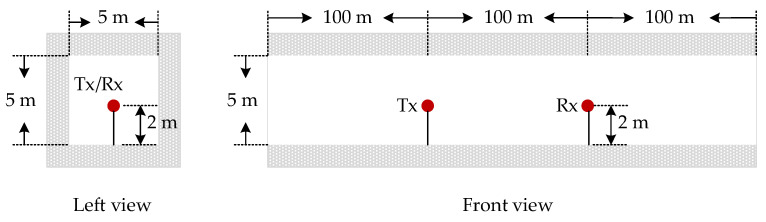
Schematic diagram of WI simulation tunnel.

In order to verify the Doppler model proposed in this paper, the RT simulation method is used in the Wireless Insite (WI) simulation software to obtain the angle and amplitude characteristics of the transmitted and received signals, and the mapping relationship of the transmitted and received signals is extracted. The 3D model of the tunnel is built using the 3D modeling software Inventor. The tunnel is a rectangular straight tunnel with a length of 300 m, a width of 5 m, and a height of 5 m. In the simulation, the signal carrier frequency is 28 GHz, and both the transmitting antenna and the receiving antenna are omnidirectional antennas, located in the center of the tunnel 100 m and 200 m away from the tunnel entrance. The distance between the antenna and the tunnel ground is 2 m, as shown in Figure 5. The tunnel structure and transmit and receive antenna parameters are listed in Table 1.

The tunnel material and simulation ray parameter settings are shown in Table 2. The tunnel material is concrete, and the parameters such as the permittivity and conductivity of the tunnel material are calibrated according to the measured data of Shanghai Metro Line 7 [10]. Moreover, the signal’s maximum reflection time in the tunnel is set to 10, its maximum scattering time is set to 2, and its transmission time is set to 0.

### 3.2. Simulation Results and Analysis

**Figure 6:**
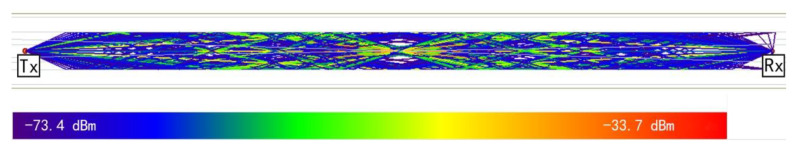
Multipath Signal Propagation Path.

**Figure 7:**
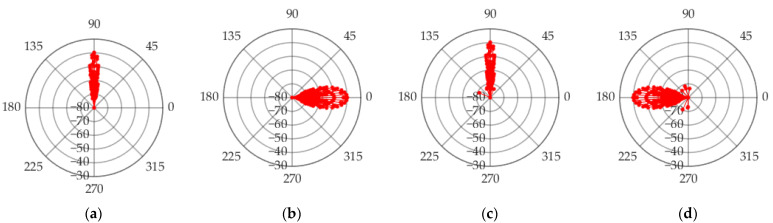
Angle and power of multipath signals (**a**) ZOD, (**b**) AOD, (**c**) ZOA, and (**d**) AOA.

The angles and powers of 250 multipath signals are obtained through the RT simulation, and the propagation paths of the multipath signals in the tunnel are shown in Figure 6. The polar coordinate form of the multipath signal angle is shown in Figure 7, in which the pitch departure angle and pitch arrival angle are concentrated around 90°, while the horizontal departure angle and horizontal arrival angle are around 0° and 180°. The angular range of the arrival angle is greater than the angular range of the departure angle. The angles and powers of the five largest energy paths in the simulation results are shown in Table 3.

The transceiver signal mapping matrix extracted from the RT simulation results can be represented as an identity matrix with 250 rows and 250 columns

(19) $$A={\left(\begin{array}{c} \begin{array}{cc} \begin{array}{cc} 1 & 0 \end{array} & \begin{array}{cc} \cdots & 0 \end{array} \end{array}\\ \begin{array}{cc} \begin{array}{cc} 0 & 1 \end{array} & \begin{array}{cc} \cdots & 0 \end{array} \end{array}\\ \begin{array}{cc} \begin{array}{cc} \vdots & \vdots \end{array} & \begin{array}{cc} \ddots & \vdots \end{array} \end{array}\\ \begin{array}{cc} \begin{array}{cc} 0 & 0 \end{array} & \begin{array}{cc} \cdots & 1 \end{array} \end{array} \end{array}\right)}_{250\times 250}$$

**Figure 8:**
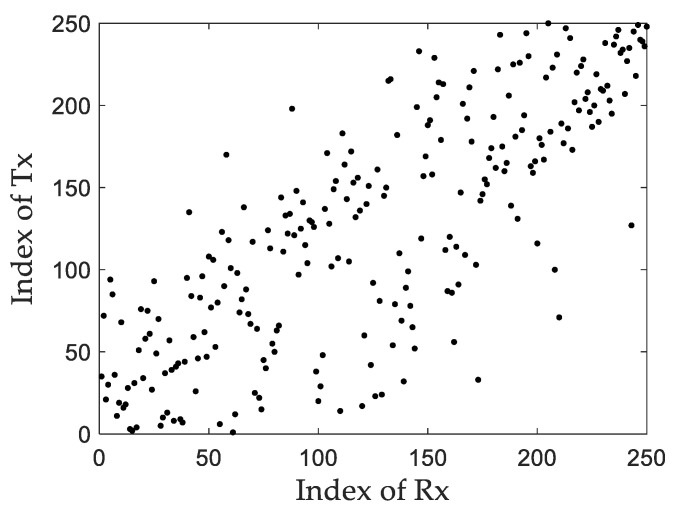
Receive and transmit signal mapping matrix.

In order to verify the spatial mirroring method proposed in this paper and the random matching method for scattered signals, after randomly arranging the transmitted signals, first determine the mapping relationship of the reflected signals between the transceivers according to the spatial mirroring principle, and then perform random matching on the unmatched signals. The complete transceiver signal mapping matrix is shown in Figure 8.

**Figure 9:**
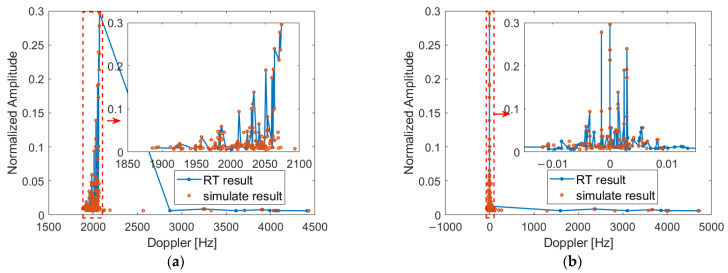
Doppler spread simulation results of T2T communication: (**a**) *v_t_* = 160 km/h, *v_r_* = 80 km/h, (**b**) *v_t_* = 160 km/h, *v_r_* = 160 km/h.

After obtaining the angle, power information, and mapping relationship of the transmitting and receiving signals, the normalized Doppler power spectrum of the transmitting and receiving antennas at different moving speeds is obtained through the Doppler model, as shown in Figure 9. The normalized amplitude in the graph is defined as the ratio of the power of each single path to the total power of the multipath. When the moving speeds of the receiving and transmitting antennas are 160 km/h and 80 km/h, the Doppler shift of the LoS signal in the RT simulation results is 2.074 KHz, and the Doppler spread is 2.516 KHz. The Doppler shift of the LoS signal is 2.074 KHz and the Doppler spread is 2.542 KHz in the simulation results of the image space method and the random matching method of scattered signals. When the receiving and transmitting antennas travel in the same direction at the same speed, the Doppler shift of the LoS signal in the RT simulation is 0 Hz, and the Doppler spread is 4.702 KHz. The Doppler shift of the LoS signal is 0 Hz and the Doppler spread is 4.783 KHz in the simulation results of the image space method and the random matching method of scattered signals. The Doppler frequency component to the right of the LoS path is the scattered signal component. The simulation results of the Doppler spread simulation model in this paper are consistent with the simulation results of RT, which verifies the availability of the Doppler spread model. The simulation results also show that in the T2T communication scenario, the Doppler spread of the signal is not only related to the antenna moving speed, but also to the departure and arrival angles. Due to the inconsistent angular ranges of the arrival and departure angles of the scattered signals, the extent of the Doppler frequency spreading to the right of the LoS path is increased.

## 4. Physical Simulation Model of T2T Communication Channel in Tunnel

In a complex propagation environment, two kinds of fading channels will be generated due to the delay spreading effect of multipath channels, namely, the frequency flat fading channel and the frequency selective fading channel. Multipath effects cause the amplitude of the received signal to shift over time when the signal bandwidth $B_{s}$ is smaller than the coherence bandwidth $B_{c}$, but the signal spectrum does not. In this case, the duration of the symbol $T_{s}$ is greater than the maximum time delay $\tau_{max}$ of multipaths, and this channel is called the flat fading channel. In a flat fading channel, the influence of the time delay on the communication system can be ignored. Existing test results show that the multipath delay in tunnel scenarios is tens of nanoseconds [11,12,13,14], that is, $\tau_{max}<T_{s}$, so the bandpass form of Equation (17) can be expressed as the product of the bandpass transmit signal $x_{p}\left(t\right)$ and the multiplicative spreading factor H(t)

(20) R(t)=Re{xp(t)H(t)}=Re{xp(t)∑i=1Iciej2πfdit}

where $x_{p}\left(t\right)=x_{p,I}\left(t\right)+jx_{p,Q}\left(t\right)$, $H\left(t\right)=\left|H\left(t\right)\right|e^{j\varphi \left(t\right)}=H_{I}\left(t\right)+jH_{Q}\left(t\right)$, then Equation (20) can be expressed as

(21) R(t)=Re{xp(t)|H(t)|ejφ(t)}=xp,I(t)HI(t)−xp,Q(t)HQ(t)

where $\varphi \left(t\right)=\tan^{-1}\left(H_{I}\left(t\right)/H_{Q}\left(t\right)\right)$ is the phase shift caused by the Doppler shift of multipaths.

**Figure 10:**
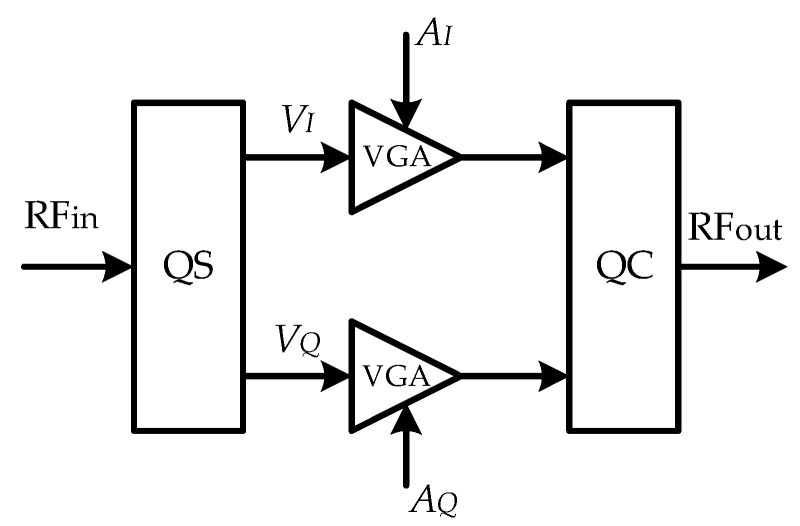
The circuit structure of IQ vector phase shifter.

**Figure 11:**
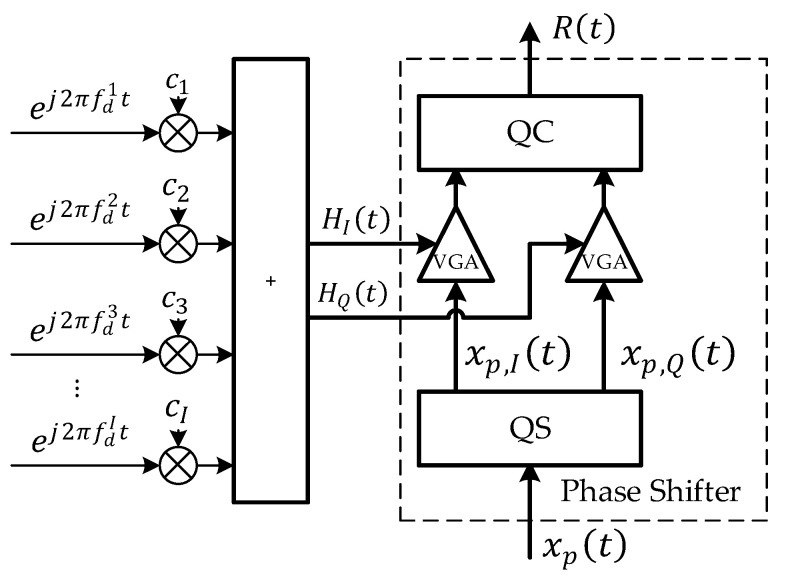
Physical simulation model of the channel.

The circuit structure of the IQ vector phase shifter [15,16] is shown in Figure 10. The phase shifter circuit consists of a quadrature splitter (QS), a variable gain amplifier (VGA), and a quadrature combiner (QC). The input RF signal $RF_{in}$ generates the in−phase component $V_{I}=RF_{in}/\sqrt{2}$ and the quadrature component $V_{Q}=jRF_{in}/\sqrt{2}$ after passing through the quadrature splitter. After $V_{I}$ and $V_{Q}$ pass through independent VGAs, they are summed in the combiner, and the output signal $RF_{out}$ is a function of the VGA gains $A_{I}$ and $A_{Q}$

(22) $$RF_{out}=A_{I}V_{I}+A_{Q}V_{Q}=A_{I}\frac{RF_{in}}{\sqrt{2}}+jA_{Q}\frac{RF_{in}}{\sqrt{2}}$$

where the gain range of VGA is {−1,1}, the amplitude of the output signal $RF_{out}$ and the input signal $RF_{in}$ remain unchanged, and phase difference $∆\varphi =\tan^{-1}\left(A_{I}/A_{Q}\right)$. Compared to (21) and (22), make $x_{p}=RF_{in}$, then $x_{p,I}=RF_{in}/\sqrt{2}$, $x_{p,Q}=jRF_{in}/\sqrt{2}$, $A_{I}=H_{I}$, $A_{Q}=-H_{Q}$. Therefore, the physical simulation of the channel can be theoretically realized by using the program-controlled IQ vector phase shifter. The physical simulation model is shown in Figure 11. In Figure 11, the IQ vector phase shifter is a program-controlled phase shifter that can be controlled in real time.

## 5. Conclusions

In this paper, the Doppler shift and Doppler spread of T2T communication in a tunnel environment are studied. Independent coordinate systems are established at the receiving and transmitting antennas. According to the angle and amplitude characteristics of the receiving and transmitting signals, the Doppler spread caused by the movement of the receiving and transmitting antennas is analyzed. The use of the mapping matrix approach to solve the matching problem of the transmitting and receiving signals is presented as an innovative solution, and two methods for obtaining the mapping matrix are described and verified. In order to verify the T2T Doppler spread simulation model proposed in this paper, the RT method is used to simulate the T2T communication channel in the tunnel, and the angle and amplitude information of the transmitted and received signals are obtained. By comparing the Doppler results of the simulation model with those of WI simulation, the correctness of the T2T Doppler spread simulation model is proved. Based on the Doppler spread model, a physical channel simulation method using an IQ vector phase shifter to complete T2T communication in a tunnel environment is proposed, which can provide a reference for the physical channel simulation of 5G mmWave T2T communication in a tunnel environment in the future.

## Figures and Tables

**Table 1:** Parametric structure of transmitted and receiving antennas in tunnel.

Parameter	Value
*f_c_* (GHz)	28
(*L, W, H*) (m)	(300,5,5)
(*x_t_*, *y_t_*, *z_t_*) (m)	(100,0,2)
(*x_r_*, *y_r_*, *z_r_*) (m)	(200,0,2)
(*v_t_*, *v_r_*) (km/h)	(160,80), (160,160)

**Table 2:** WI simulation parameter settings.

**Tunnel Parameters**	**Material**	**Permittivity**	**Conductivity (S/m)**	**Thickness (m)**	**Roughness (m)**
	Concrete	5.31	0.48	0.5	0.005
**Ray Parameters**	**Reflections Times**	**Transmission Times**	**Scattering Times**	**Interval of Rays**	**Number of Rays**
	10	0	2	0.25°	250

**Table 3:** Angle and power of 5 maximum energy paths.

Number of Multipath	$\phi_{AOD\;}\left({}^{\circ}\right)$	$\theta_{ZOD\;}\left({}^{\circ}\right)$	$\phi_{AOA}\;\left({}^{\circ}\right)$	$\theta_{ZOA}\;\left({}^{\circ}\right)$	$P_{r}\;\left(dBm\right)$
1	0	90	−180	90	−39.8109
2	−2.86241	90	−177.138	90	−40.3604
3	2.86241	90	177.138	90	−40.3604
4	−5.71059	90	174.289	90	−41.6511
5	5.71059	90	−174.289	90	−41.6511

## Data Availability

Not applicable.
